# A Lethal Complication of Endoscopic Therapy: Duodenal Intramural Hematoma

**DOI:** 10.1155/2015/201675

**Published:** 2015-11-30

**Authors:** Turan Calhan, Abdurrahman Sahin, Resul Kahraman, Barış Soydaş, Ali Tosun, Egemen Cebeci

**Affiliations:** ^1^Department of Gastroenterology, Mehmet Akif İnan Training and Research Hospital, Şanlıurfa, Turkey; ^2^Department of Gastroenterology, Umraniye Training and Research Hospital, Istanbul, Turkey; ^3^Department of Radiology, Mehmet Akif İnan Training and Research Hospital, Şanlıurfa, Turkey; ^4^Department of Nephrology, Okmeydanı Training and Research Hospital, Istanbul, Turkey

## Abstract

Duodenal intramural hematoma (DIH) usually occurs in childhood and young adults following blunt abdominal trauma. It may also develop in the presence of coagulation disorders and may rarely be an iatrogenic outcome of endoscopic procedures. Management of DIH is usually a conservative approach. A case of intramural duodenal hematoma that developed following endoscopic epinephrine sclerotherapy and/or argon plasma coagulation and that was nonresponsive to conservative therapy in a patient with chronic renal failure who died from sepsis is being discussed in this report. Clinicians should be aware of such possible complications after endoscopic hemostasis in patients with coagulation disorders.

## 1. Introduction

Endoscopic hemostatic procedures such as local injection of epinephrine, polidocanol, and fibrin tissue adhesive onto the mucosa, argon plasma coagulation (APC), and hemoclipping are commonly used for the treatment of bleeding ulcers, instead of the open surgical approaches used in the past [1]. Although the risks are usually considered to be minimal, there are reports describing that duodenal intramural hematomas may develop as a complication after diagnostic or therapeutic endoscopy, especially in patients susceptible to hemorrhage, such as those with chronic renal disease [2]. Herein we discuss a case of duodenal intramural hematoma (DIH), complicated by acute pancreatitis, obstructive jaundice, and sepsis, which developed after endoscopic epinephrine sclerotherapy and/or APC and which was nonresponsive to conservative therapy in a patient with chronic renal failure secondary to hypertensive nephrosclerosis.

## 2. Case Presentation

**Figure 1:**
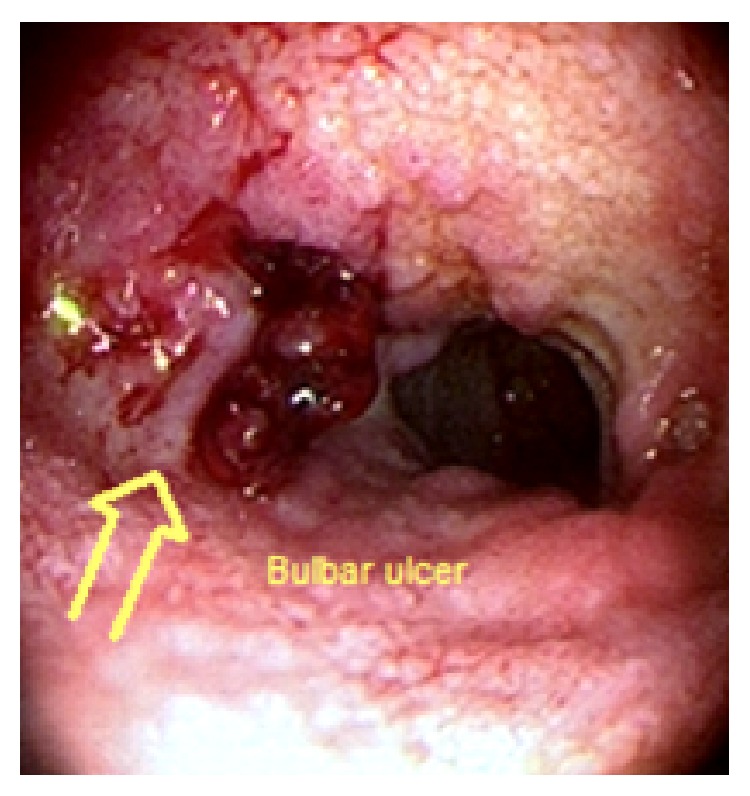
Bleeding ulcer covered with fresh red clot at the anterior wall of the bulb duodenal segment II opening. There was oozing bleeding at the ulcer when the clot was eliminated (prior to endoscopic procedure).

**Figure 2:**
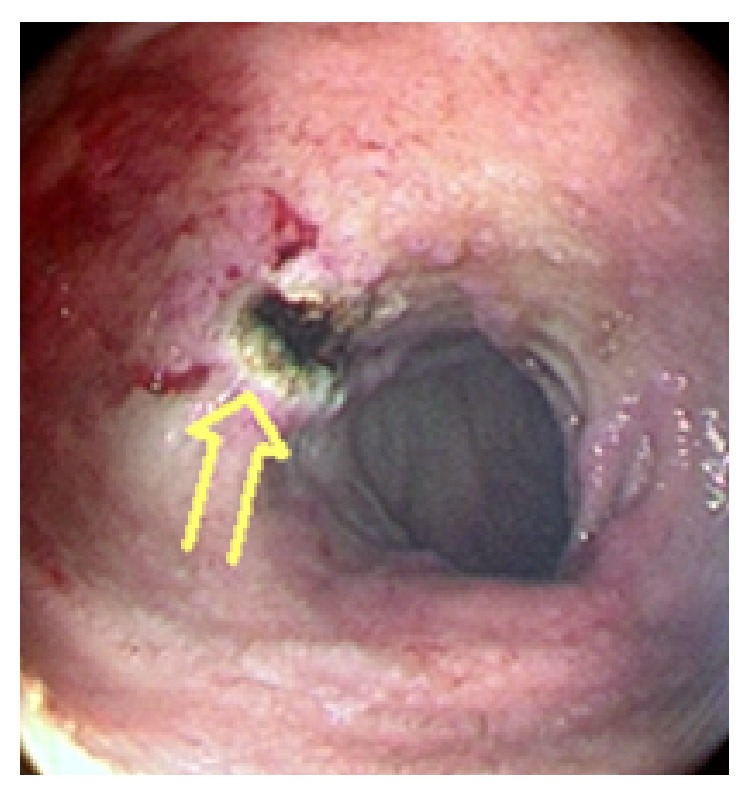
The image following endoscopic epinephrine sclerotherapy plus endoscopic argon plasma coagulation (arrow) procedure for bleeding bulbar ulcer shows complete cessation of bleeding.

**Figure 3:**
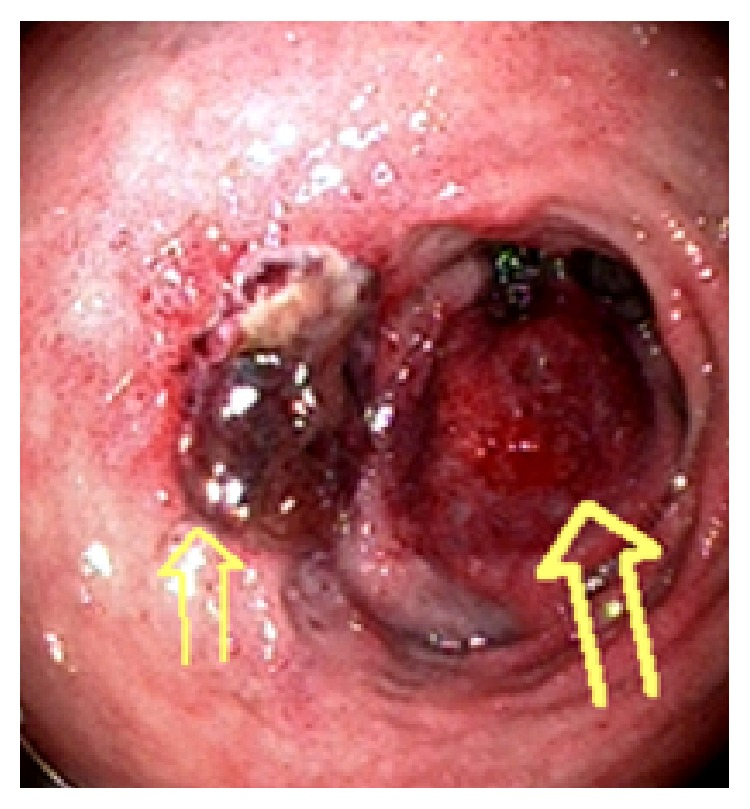
Intramural duodenal hematoma at the posterior duodenal wall not causing complete luminal obstruction (large arrow), which developed 36 hours after endoscopic procedure. The ulcer appears stable following endoscopic treatment (small arrow).

**Figure 4:**
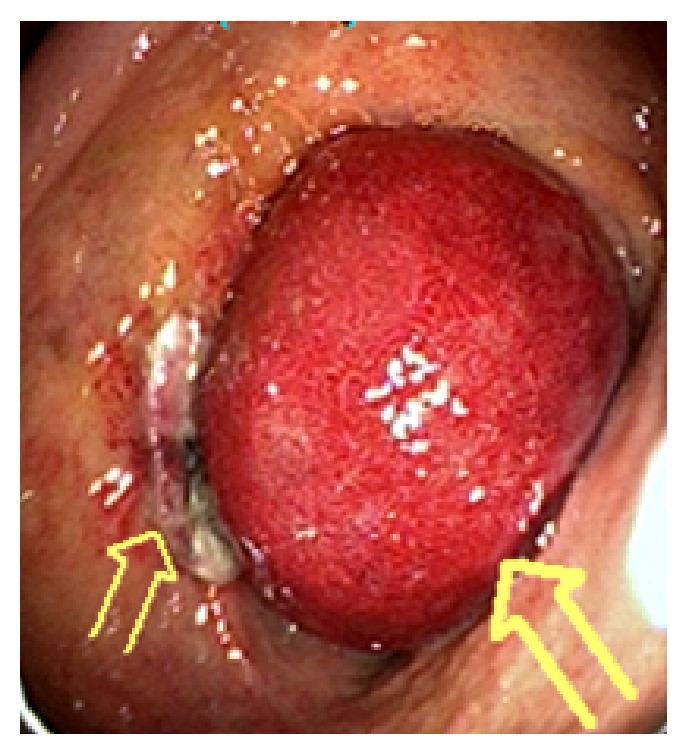
Giant intramural duodenal hematoma causing complete luminal obstruction 60 hours after the endoscopic procedure (large arrow). Bulbar ulcer appears stable (small arrow).

**Figure 5:**
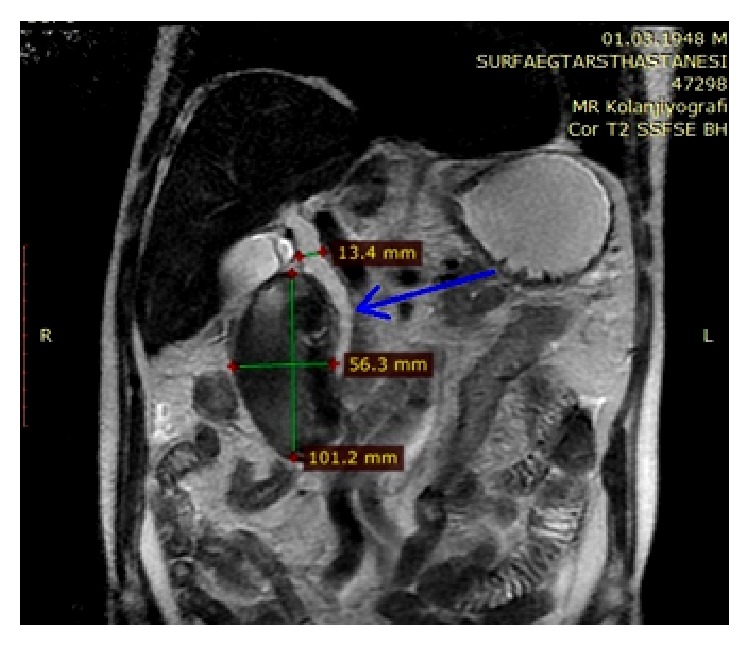
Coronal T2A magnetic resonance imaging. The image shows a hematoma with a diameter of approximately 10.1 × 5.6 cm. Bile ducts are dilated (common bile duct (CBD) diameter 13.4 mm). Distally, pressure caused by the hematoma on the CBD and reduced CBD diameter is apparent (blue arrow).

**Figure 6:**
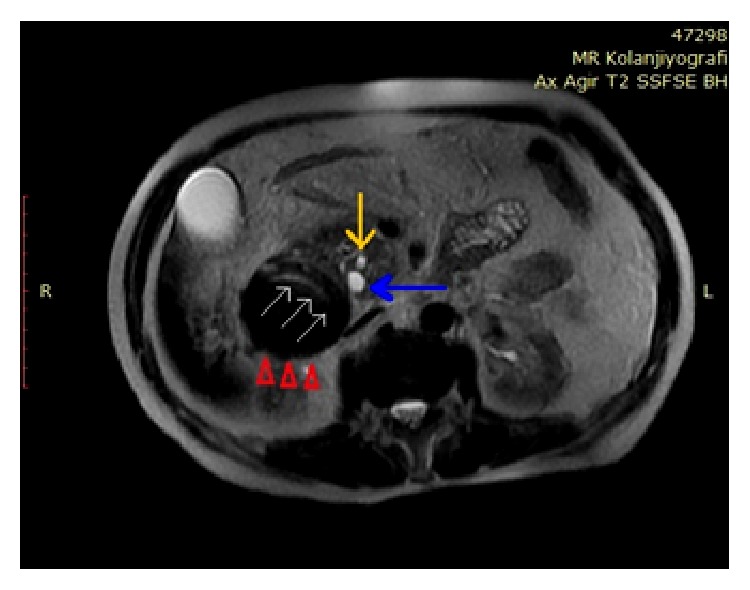
T2A magnetic resonance imaging from the axial plane. Intramural duodenal hematoma adjacent to the pancreas head (red arrow heads). Duodenal lumen appears to be pushed forward due to the hematoma with significant narrowing of its lumen (white arrows). Common bile duct (blue arrow) and pancreatic duct (yellow arrow) are dilated due to pressure caused by the hematoma.

**Figure 7:**
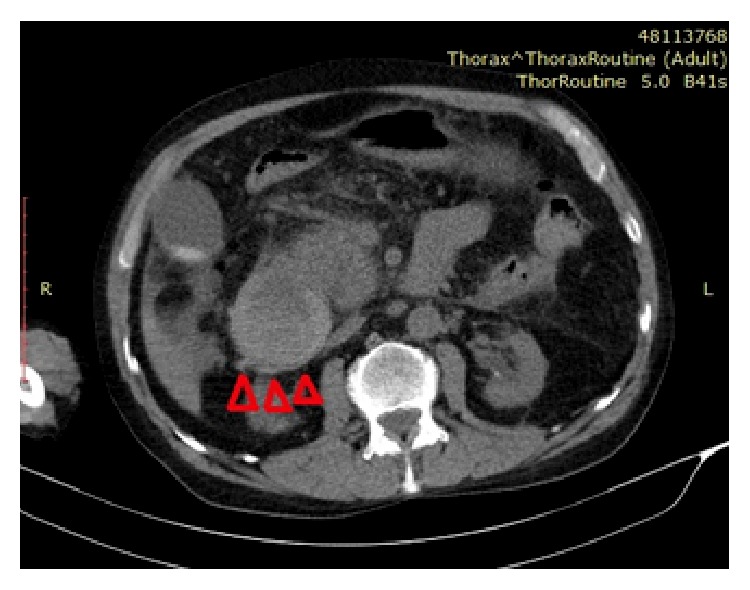
Computerized tomography image from the same axial plane as magnetic resonance imaging (Figure 6), showing intramural hematoma (red arrow heads).

A 65-year-old male patient was admitted to the clinic with weakness and dark stool persisting for the last 3-4 days. He had hypertension (HT) and chronic renal failure (CRF) and no habit of smoking or alcohol consumption. He had no history of using anticoagulants or antiaggregant drugs. On physical examination, the patient appeared pale and tired. His skin turgor and tone were decreased. Blood pressure was 90/60 mmHg, pulse rate 120 beats/min, and body temperature 36.4°C. Lung examination was normal. Abdominal examination demonstrated mild epigastric tenderness and melena was identified by rectal examination. Initial laboratory values were as follows: glucose: 107 mg/dL, urea: 361 mg/dL, creatinine: 7.1 mg/dL, sodium: 139 mEq/L, potassium: 3.86 mEq/L, leukocyte: 21.300/mm^3^, hemoglobin: 7.8 g/dL, platelets: 100.000/mm^3^, prothrombin time: 12 sec, and INR: 1.05. Serum transaminase, bilirubin, and amylase-lipase values were within normal ranges. Crystalloid and colloid fluid replacement were promptly given intravenously, parenteral proton-pump inhibitor was initiated, and four units of erythrocyte suspension was administered. Following hemodynamic stabilization, the patient underwent upper gastrointestinal endoscopy (UGIE). A 9 mm sized ulcer, covered with fresh red clot, was observed at the anterior wall of the bulb. The clot was removed and oozing bleeding was seen at the base of the ulcer; epinephrine was then injected into the areas adjacent to the ulcer. A vascular lesion at the ulcer base was coagulated with argon plasma coagulation (APC) during the same session (Figures 1 and 2). Although there had been no evidence of clinical bleeding (no hematemesis or melena), hemoglobin and hematocrit values declined approximately 36 hours later from this intervention, and a second UGIE was carried out. On endoscopic examination, there was no bleeding from the ulcer. However, the posterior wall mucosa of the second part of the duodenum, at approximately 2 cm distal to the ulcer, was erythematous with no obstruction (Figure 3). Postoperatively the patient developed nausea, vomiting, abdominal pain, and jaundice after the second endoscopic assessment. Serum total bilirubin and direct bilirubin values increased up to 23 mg/dL and 19 mg/dL, respectively. Plasma amylase level was 1389 U/L, and lipase level was 991 U/L. At the third day of admission, a final UGIE was performed to investigate the possible presence of a hematoma. The ulcer was stable but the mucosa of the second part of the duodenum was erythematous and was also significantly protruded towards the lumen, resulting in complete obstruction (Figure 4). Upper abdominal magnetic resonance imaging (MRI) and computerized tomography (CT) demonstrated a mass with size 5.6 × 10.1 cm at the section corresponding to the second and third parts of the duodenum, consistent with an intramural hematoma causing complete obstruction of the lumen. The lesion compressed the bile ducts and the pancreatic duct resulting in dilation of the common bile duct and main pancreatic duct (Figures 5, 6, and 7). Decompression with a nasogastric tube was performed and a conservative treatment modality was attempted (total parenteral nutrition, intravenous fluid, and careful observation). However, the patient did not respond to conservative treatment and his overall condition deteriorated rapidly with signs of sepsis developing. In response, ultrasonography-guided percutaneous hematoma drainage was performed. The patient's clinical parameters did not improve although the hematoma was drained. The patient died because of sepsis, septic shock, and multiorgan failure.

## 3. Discussion

Duodenal intramural hematoma is an unusual condition in adults [3]. Blunt abdominal trauma is involved in the etiology of most of the cases (more than 80%) [4]. Nontraumatic DIHs usually result from anticoagulant treatment, blood dyscrasia, pancreatic diseases, intestinal arterial aneurysm rupture, and endoscopic procedures on the duodenal mucosa [5]. DIHs following endoscopic intervention (e.g., biopsy, sclerotherapy, and APC) are extremely rare [6, 7]. DIHs usually occur at the posterior wall of the duodenum fixed with the retroperitoneum in front of the vertebral colon, a region that is rich in submucosal vascular structure [4]. It has been reported that hematomas may particularly develop more often in the presence of conditions that predispose to bleeding, including hepatic cirrhosis and renal failure [1, 8]. Treatment of DIHs is usually conservative and recovery with conservative treatment has been shown especially in childhood and in cases with gastric outlet obstruction [9]. When conservative treatment is not effective, surgical drainage choices of ultrasound- or CT-guided drainage (laparotomy, laparoscopic drainage) are used [10]. Additionally, endoscopic incision and drainage techniques are used in the treatment as a more comfortable method [11]. There have been reports in the literature of DIH cases following both APC and endoscopic epinephrine sclerotherapy [1, 12]. Epinephrine sclerotherapy followed by APC during a single session was performed to our patient. The patient had CRF and unexplained thrombocytopenia (100.000/mm^3^) as risk factors. The etiology of hemorrhagic diathesis in CRF is multifactorial. Several factors, including platelet dysfunction, anemia, and inadequate renal excretion of drugs, are involved [13]. We believe that DIH was caused rather by sclerotherapy in the current case. However, it is likely that APC could have caused or contributed to the condition.

Following the observation of endoscopic findings suggestive of DIH, imaging methods were performed to confirm the diagnosis. Although abdominal ultrasonography was performed initially, chosen for its noninvasive nature and convenience, it was suboptimal due to abdominal gas and demonstrated an abdominal mass with indefinite margins. Abdominal CT was performed to determine the location of the mass, to rule out possible perforation and to view the size of the mass. Simultaneous MR cholangiography was also undertaken to demonstrate the dilatation of the bile ducts. After establishing the DIH diagnosis with imaging techniques, nasogastric decompression was performed and conservative treatment (total parenteral nutrition, intravenous fluid, and careful observation) was attempted. However, a giant hematoma leading to icterus and sepsis, complete duodenal obstruction, and acute pancreatitis developed at 60 hours. He was considered unresponsive to conservative treatment and ultrasonography-guided percutaneous hematoma drainage was performed. The patient unfortunately died on the next day due to sepsis, septic shock, and multiorgan failure.

There have been reports in the literature describing failure with conservative treatment in cases of DIH that developed following endoscopic sclerotherapy complicated with jaundice and acute pancreatitis [2], even including fatal outcome [14]. Conservative treatment may be followed for patients without complete duodenal obstruction and/or complications. However, in cases involving complete intestinal obstruction and/or pressure on adjacent organs, we believe that the hematoma should be identified as early as possible and evacuated dynamically both internally and externally to avoid fatal outcomes (acute pancreatitis, obstructive jaundice, aspiration pneumonia, and sepsis/septic shock). As a minimally invasive treatment choice, ultrasound- or CT-guided drainage, laparotomy, laparoscopic drainage, and endoscopic incision and drainage should be considered earlier for such cases.

In conclusion, it should be kept in mind that endoscopic hemostatic procedures in the presence of conditions predisposing patients to bleeding (e.g., chronic renal failure and hepatic cirrhosis) may lead to complications, and any deterioration should prompt a reassessment, utilizing imaging methods as necessary. Intramural hematoma should be considered in differential diagnosis for patients with no clinical evidence of bleeding (no hematemesis or melena) but with reduced hematocrit levels after endoscopic intervention. Clinicians should not insist on maintaining the treatment when complications such as complete luminal obstruction, acute pancreatitis secondary to pressure on the adjacent organs, and obstructive jaundice develop and should consider other treatment choices earlier.
